# The Cardio-Renal-Hepatic Axis in MASLD. Role of BDNF, NGF, and Metabolic Cross-Talk

**DOI:** 10.1007/s11906-026-01388-1

**Published:** 2026-09-04

**Authors:** Lucy C. Taylor, Michael I. Adenawoola, Claire B. Wingfield, Maren K. Nadolski, David E. Stec

**Affiliations:** Department of Physiology & Biophysics, Cardiovascular-Renal Research Center, Cardiorenal, and Metabolic Diseases Research Center, University of Mississippi Medical Center, Jackson, MS 39216 USA

**Keywords:** Hepatokines, Heart, Hypertension, Cardiac function, Kidney

## Abstract

**Purpose of Review:**

To evaluate the crosstalk between the liver, heart, and kidneys in metabolic dysfunction-associated steatotic liver disease (MASLD) and metabolic dysfunction-associated steatohepatitis (MASH), highlighting the distinct regulatory roles of non-traditional hepatokines, and to assess shared therapeutic targets and molecular mechanisms driving cardiorenal complications in MASLD patients.

**Recent Findings:**

Emerging evidence highlights that hepatic lipid accumulation drives cardiac damage, which manifests as adverse remodeling, diastolic dysfunction, and coronary microvascular dysfunction. These abnormalities are mediated primarily by systemic lipotoxicity, inflammation of epicardial adipose tissue, and a pro-thrombotic environment. Mechanistically, altered hepatokine signaling mediates systemic cross-talk. Specifically, the downregulation of cardioprotective and renoprotective brain-derived neurotrophic factor (BDNF) and the dual metabolic roles of nerve growth factor (NGF), fibroblast growth factor (FGF) 19, and FGF21 accelerate the progression of both heart failure and chronic kidney disease (CKD). Concurrently, metabolic therapies such as glucagon-like peptide-1 (GLP-1) receptor agonists, sodium-glucose cotransporter-2 (SGLT2) inhibitors, and peroxisome proliferator-activated receptor (PPAR) agonists demonstrate significant efficacy in ameliorating steatosis, inflammation, and overall metabolic outcomes.

**Summary:**

MASLD acts as a systemic driver of multi-organ dysfunction within the cardio-renal-hepatic axis. Profiling specific hepatokine signatures offers a promising frontier for identifying novel therapeutic targets and biomarkers of disease acceleration. Furthermore, while current metabolic therapies show remarkable potential, rigorous clinical testing remains critical to definitively determine whether their cardiovascular and renal benefits stem from direct tissue-specific protective mechanisms or secondary metabolic improvements.

## Introduction

**Fig. 1:**
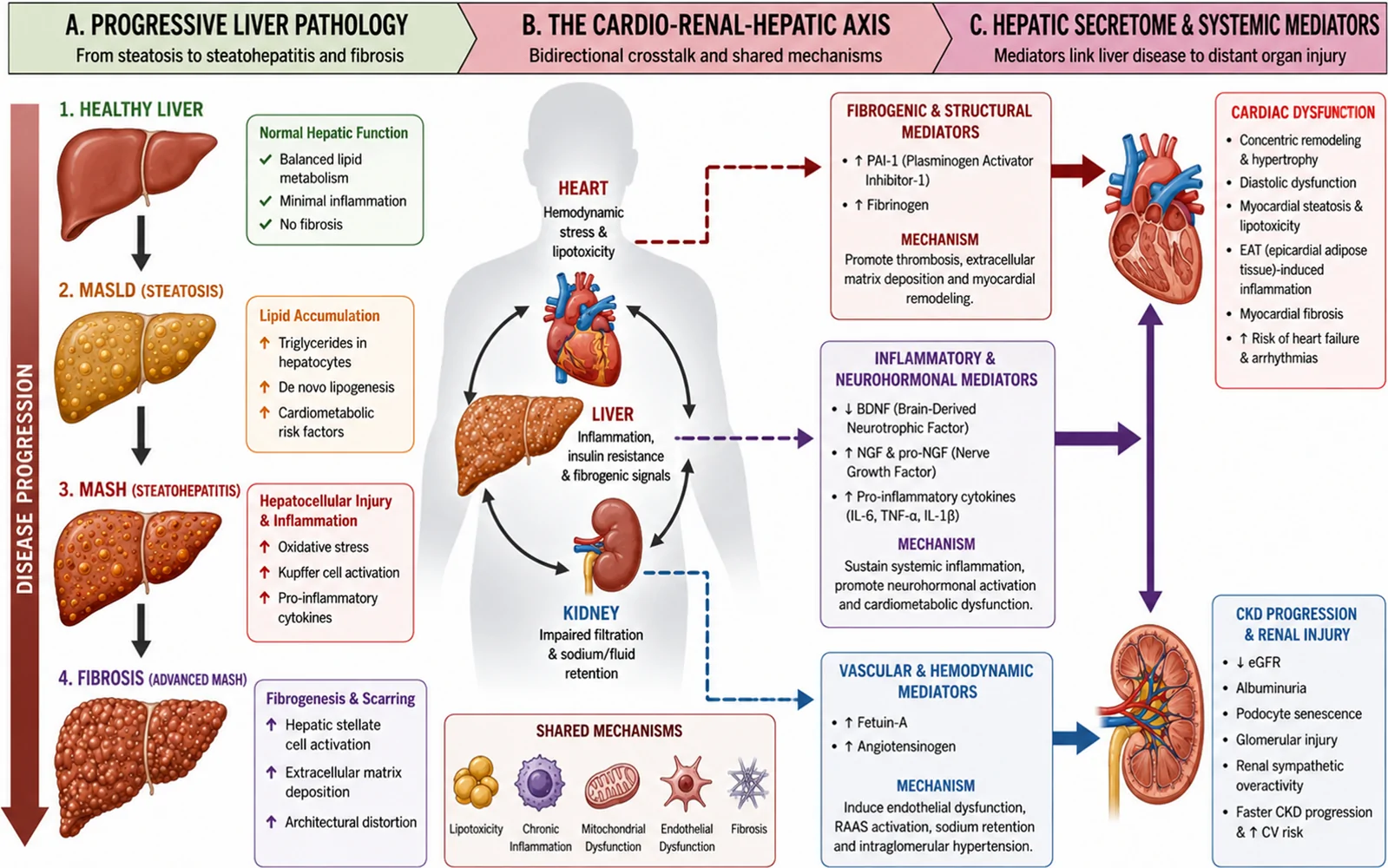
The Cardio-Renal-Hepatic Axis in MASLD. **A** Progressive Liver Pathology: The transition from a healthy baseline to MASLD (lipid accumulation) and advanced MASH (chronic inflammation and fibrosis. **B** The Cardio-Renal-Hepatic Axis: Multi-organ communication defines the cardiorenal-metabolic syndrome continuum, where injury to one organ exacerbates dysfunction in the others. **C** Hepatokines & Systemic Mediators: Branching pathways through which the diseased liver releases hepatokines into circulation: (top) elevated PAI-1 and Fibrinogen establish a hypercoagulable, pro-thrombotic state; (middle) shared mediators targeting both organs, including decreased BDNF, increased NGF/pro-NGF, and elevated pro-inflammatory cytokines; (bottom) elevated Fetuin-A and angiotensinogen drive localized renal stress and RAAS activation. Release of these systemic mediators culminates in distinct clinical phenotypes in the cardiovascular system and kidneys. Figure was created with BioRender

The recent redefinition of hepatic steatosis as metabolic dysfunction-associated steatotic liver disease (MASLD) emphasizes the intrinsic link between the liver and systemic metabolic health. Specifically, MASLD encompasses the broader spectrum of steatotic liver disease, characterized by the accumulation of excess lipids in the liver in the presence of at least one cardiometabolic risk factor, such as obesity or type 2 diabetes mellitus (T2DM) [1]. Disease progression from MASLD to metabolic dysfunction-associated steatohepatitis (MASH) is characterized by hepatocyte ballooning, oxidative stress, immune cell infiltration, and progressive hepatic fibrosis. However, the clinical impact of MASLD extends far beyond the hepatic parenchyma. MASLD is increasingly recognized as a systemic driver of multi-organ dysfunction. Specifically, the cardio-renal-hepatic axis represents a critical pathway in which hepatic pathology directly influences cardiac and renal function [2]. Clinical data consistently demonstrate that MASLD patients are at a significantly increased risk for cardiovascular disease (CVD) and chronic kidney disease (CKD), often independent of traditional risk factors like hypertension or dyslipidemia [3–5]. The primary cause of death in MASLD is cardiovascular disease (CVD), which frequently occurs before liver-related complications [6, 7]. Epidemiological studies consistently link MASLD to an elevated risk of coronary artery disease, heart failure, atrial fibrillation, and stroke [8]. A steady rise in cardiovascular risk corresponds with the transition from simple steatosis to fibrosis and cirrhosis (Fig. 1), indicating that the severity of liver disease, especially fibrosis, is a significant predictor of cardiac outcomes. Hence, MASLD is an important component of the cardiovascular-kidney-metabolic (CKM) syndrome continuum.

Cross-talk between organs is evident in several clinical pathologies, such as hepatorenal and cardiorenal syndromes. In hepatorenal syndrome, severe liver dysfunction resulting from cirrhosis, alcoholic hepatitis, or liver injury is believed to alter renal blood flow and result in renal failure. Cardiorenal syndrome occurs when injury to the heart or kidney affects the subsequent function of the other organ. Cardiorenal syndrome is mediated by alterations in hemodynamic factors, systemic inflammation, increased oxidative stress, and neurohormonal activation. Recent evidence has emerged linking increased hepatic steatosis to alterations in cardiac function. Despite this emerging relationship, the factors underlying alterations in cardiac function in response to increased hepatic fat levels remain unknown. This review will summarize the latest knowledge on cardiac structural and functional changes resulting from hepatic steatosis, as well as the potential hepatokines that may mediate these effects.

### Structural Cardiac Changes in MASLD

Multiple imaging and human cohort studies demonstrate that MASLD is associated with adverse cardiac remodeling, even in asymptomatic individuals [9–11]. These changes include increased left ventricular (LV) mass, relative wall thickness (concentric remodeling), and altered LV geometry. They are closely associated with the degree of hepatic steatosis and the amount of fibrosis, indicating a dose-dependent interaction between the liver and the heart. Chronic hemodynamic stress, systemic inflammation, and metabolic dysregulation are thought to cause concentric remodeling in MASLD. These factors raise myocardial workload and stimulate hypertrophic signaling pathways [12]. In addition to structural remodeling, MASLD is associated with ectopic lipid deposition in the heart, leading to myocardial steatosis. Cardiomyocytes absorb excess circulating free fatty acids (FFAs), which are produced by the liver and insulin-resistant adipose tissue. This leads to oxidative stress, mitochondrial dysfunction, and the accumulation of lipotoxic intermediates, such as ceramides and diacylglycerols [13]. These mechanisms directly link hepatic lipid dysregulation to cardiac damage by impairing cardiomyocyte contractility and promoting apoptosis [14].

### Functional Cardiac Changes in MASLD

Multiple functional cardiac abnormalities are strongly linked to hepatic steatosis, ranging from impaired diastolic relaxation to microvascular insufficiency and elevated filling pressures. The hallmark features of diastolic dysfunction in MASLD stem from an integrated pathophysiology involving myocardial fibrosis, extracellular matrix (ECM) remodeling, lipotoxicity-induced calcium dysregulation, coronary microvascular dysfunction, and epicardial adipose tissue (EAT)-driven inflammation [14–19].

Subclinical diastolic dysfunction, characterized by impaired ventricular relaxation and elevated filling pressures, is one of the earliest detectable functional deficits to occur in human cohort studies of MASLD. This impairment frequently precedes overt heart failure [15] and is strongly associated with heart failure with preserved ejection fraction (HFpEF) [16], a condition driven by myocardial stiffness and metabolic inflammation [12]. Diastolic dysfunction in MASLD is driven by interconnected metabolic, inflammatory, and cardiovascular alterations that promote myocardial stiffness and impaired relaxation. A central mechanism is systemic low-grade inflammation and myocardial fibrosis [17]. Chronic hepatic inflammation leads to elevated circulating levels of tumor necrosis factor-alpha (TNF-α) and interleukin-6 (IL-6), which activate cardiac fibroblasts and stimulate collagen deposition within the myocardium. This fibrotic remodeling increases ventricular stiffness and impairs diastolic filling [18, 19]. Myocardial fibrosis and unfavorable cardiac remodeling are closely associated with diastolic dysfunction in MASLD. Increased myocardial stiffness and impaired ventricular relaxation result from profibrotic signaling pathways, such as those driven by transforming growth factor-β (TGF-β), triggered by systemic metabolic stressors, including insulin resistance and lipotoxicity. Clinical evidence showing that people with MASLD have subclinical myocardial remodeling and diastolic dysfunction irrespective of overt cardiovascular illness supports these structural and functional changes [9, 11, 15, 20].

At the cellular level, toxic lipid intermediates (ceramides and diacylglycerols) derived from both lipolytic and visceral adipose tissue, as well as from hepatic de novo lipogenesis, impair cardiomyocyte calcium reuptake, prolonging cytosolic calcium transients and delaying myocardial relaxation. In addition, coronary microvascular dysfunction exacerbates this phenotype. In patients with MASLD, systemic oxidative stress and circulating proinflammatory cytokines diminish endothelial nitric oxide synthase activity, reduce nitric oxide availability, and increase coronary vascular resistance, culminating in subclinical microvascular ischemia [19].

Epicardial adipose tissue (EAT) is the visceral fat depot situated between the outer myocardial wall and the visceral pericardium, sharing an unobstructed microvascular and stromal interface with the underlying myocardium [19, 21]. Under normal physiological conditions, EAT serves cardioprotective functions, including local lipid buffering, thermoregulation, and mechanical cushioning [21]. In MASLD, however, chronic metabolic overload induces marked EAT hypertrophy, local hypoxia, macrophage infiltration, and a phenotypic switch toward a pro-inflammatory secretome [19, 21, 22]. Human studies demonstrate that greater EAT burden is linked to both hepatic steatosis and markers of subclinical cardiac dysfunction, supporting a potential paracrine interaction between EAT and the adjacent myocardium that may contribute to myocardial remodeling and impaired relaxation [23]. Clinical echocardiographic and cardiac magnetic resonance studies confirm a clear association between liver illness and myocardial failure when structural cardiac abnormalities like left ventricular hypertrophy and concentric remodeling are combined with functional deficiencies like diastolic dysfunction [10].

MASLD is characterized by systemic metabolic dysregulation that extends to hemostatic and adipose tissue pathways, thereby promoting cardiovascular disease through defined molecular mechanisms. Among these, the development of a pro-thrombotic state and the inflammatory activation of epicardial adipose tissue (EAT) are key contributors. These processes are closely linked to insulin resistance, visceral adiposity, and chronic low-grade inflammation, which are central features of MASLD.

### Molecular Mechanisms Linking MASLD to Cardiac Dysfunction

Hepatokines are proteins and signaling peptides synthesized and secreted by hepatocytes that exert autocrine, paracrine, and endocrine actions across organ systems. In MASLD, metabolic stress alters hepatokine secretion, transforming the hepatic secretome into a major driver of cardiac and renal dysfunction.

While classically characterized in the central nervous system, BDNF is also produced by hepatocytes and cardiomyocytes in response to metabolic cues, exercise, and energy-sensing cascades. In cardiovascular tissues, BDNF binds its high-affinity receptor, tropomyosin receptor kinase B (TrkB), activating pro-survival PI3K/Akt and mitogen-activated protein kinase (MAPK) pathways that support cardiomyocyte contractility and mitochondrial bioenergetics. In human study cohorts, low circulating BDNF levels are associated with an increased risk of heart failure and major cardiovascular events [24–26]. BDNF acts as a survival factor for cardiomyocytes, and its repression in MASLD represents a key vulnerability for the heart [27].

Hepatic dysfunction, specifically the loss of PPARα as seen in MASLD models, leads to a significant reduction in TrkB activation. BDNF/TrkB signaling is essential for normal cardiac contraction, relaxation, and survival; a deficiency in this signaling pathway induces cardiomyocyte apoptosis and contributes to the structural thinning of cardiac walls [28]. In the hepatic parenchyma, BDNF/TrkB signaling orchestrates catabolic pathways that safeguard against metabolic insult. The molecular transition from health to steatosis is largely dictated by BDNF’s ability to activate the liver AMP-activated protein kinase (AMPK) signaling pathway [16], which promotes the ß-oxidation of free fatty acids and reduces hepatic triglyceride content by suppressing fatty acid synthase activity [29]. Additionally, BDNF signaling limits hepatic glucose output and improves systemic insulin resistance by activating the cAMP response element-binding protein (CREB) pathway, which downregulates the transcription of key gluconeogenic enzymes such as phosphoenolpyruvate carboxykinase (PEPCK) [30]. By mitigating hepatic dyslipidemia and systemic hyperglycemia, this pathway ultimately reduces the lipotoxic and glucotoxic burden on the myocardium, representing a primary cardioprotective mechanism within this axis.

A breakdown in BDNF signaling can characterize the progression of MASLD to MASH. A critical molecular switch in this process involves TrkB.T1, a truncated isoform of TrkB lacking the catalytic kinase domain [31]. Clinical analyses of human liver biopsies demonstrate that TrkB.T1 expression is elevated in patients with advanced MASH, strongly correlating with markers of inflammation and necroptotic cell death. These findings were mirrored in animal models, in which chronic metabolic stress drives accumulation of TrkB.T1 on the hepatocyte membrane. This isoform acts as a negative regulator, preventing functional BDNF signaling while actively promoting inflammatory cascades and stress-induced apoptosis. Treatment with exogenous BDNF can rescue this phenotype in mice by triggering the endocytosis and lysosomal degradation of TrkB.T1, thereby clearing the pro-inflammatory signal and restoring hepatic proteostasis [31]*.* Conversely, Hattori et al. observed elevated circulating BDNF in early-stage MASLD patients compared to healthy controls, likely reflecting the liver-heart axis attempting to counteract advancing metabolic stress before the signaling architecture completely fails [32].

The pro-thrombotic phenotype in MASLD is heavily driven by plasminogen activator inhibitor-1 (PAI-1), a serine protease inhibitor that functions as a hepatokine released in response to hyperinsulinemia, lipotoxicity, and pro-inflammatory cytokines [33]. PAI-1inhibits the function of tissue plasminogen activator (tPA) and urokinase, thereby suppressing fibrinolysis, the enzymatic process responsible for degrading fibrin blood clots. Increased PAI-1 levels have been regularly noted in disorders marked by insulin resistance and obesity, such as MASLD, and are associated with heightened cardiovascular risk [21]. PAI-1 is synthesized by various cell types pertinent to MASLD pathophysiology, including hepatocytes, adipocytes, endothelial cells, and platelets. Its expression is augmented by pro-inflammatory cytokines such as TNF-α and IL-6, as well as by hyperinsulinemia. This results in hypofibrinolysis, characterized by decreased fibrinolysis and increased thrombus persistence. MASLD is also associated with elevated fibrinogen levels, a crucial coagulation factor produced in the liver, and decreased fibrinolysis. In addition to increasing platelet aggregation and blood viscosity, fibrinogen aids clot formation by converting to fibrin [33]. The risk of atherothrombotic events like myocardial infarction and stroke is increased by elevated fibrinogen levels, which are frequently seen in inflammatory and metabolic diseases and further contribute to a hypercoagulable state [22].

Epicardial adipose tissue (EAT) has emerged as a critical mediator of cardiac dysfunction in MASLD due to the adipokines and cytokins that it can release. EAT is a visceral fat depot located between the myocardium and the visceral pericardium and is distinguished by its direct anatomical and functional continuity with the myocardium and coronary arteries. Under physiological conditions, EAT serves protective roles, including energy supply and thermoregulation, lipid buffering, and mechanical protection. However, in metabolic disorders such as MASLD, EAT undergoes expansion and functional transformation into a pro-inflammatory and pro-atherogenic tissue [34]. Increased release of inflammatory cytokines and adipokines, such as TNF-α, IL-6, and monocyte chemoattractant protein-1 (MCP-1), along with increased oxidative stress, are characteristics of this dysfunctional EAT [35]. These mediators can directly diffuse into nearby cardiac tissue and coronary arteries via paracrine and exocrine signaling pathways, as the lack of a fascial barrier promotes local inflammation and vascular injury [36]. The main characteristics of cardiovascular disease linked to MASLD include coronary endothelial dysfunction, atherosclerosis, and myocardial fibrosis, all of which are influenced by these processes.

Furthermore, EAT-derived inflammatory mediators contribute to lipotoxicity and oxidative stress in cardiomyocytes (Fig. 1), impairing cellular metabolism and contractile function. EAT has also been implicated in the progression and destabilization of atherosclerotic plaques, thereby increasing the risk of acute coronary syndromes [37]. Clinical and imaging studies have demonstrated associations between increased EAT volume and adverse cardiovascular outcomes, including coronary artery disease, atrial fibrillation, and heart failure [34]. There is a molecular connection between EAT inflammation and the pro-thrombotic state. EAT and other adipose depots produce inflammatory cytokines that can further increase PAI-1 expression, aggravating hypofibrinolysis and strengthening the prothrombotic milieu (Fig. 1). As a result, there is a feedback loop in which inflammation promotes aberrant coagulation, which, in turn, leads to vascular damage and disease progression. A hypercoagulable, hypofibrinolytic state is established by elevated PAI-1 and fibrinogen levels, and the heart and coronary arteries are directly harmed by inflamed epicardial adipose tissue [38]. These pathways offer a biologically tenable, empirically supported theory linking cardiovascular pathophysiology to hepatic metabolic abnormalities.

NG performs essential functions within the cardiovascular system that go well beyond its traditional role in neural regulation. NGF’s effects are primarily mediated through its high-affinity receptor, tropomyosin receptor kinase A (TrkA), which promotes trophic and pro-survival signals, and its low-affinity receptor, p75 neurotrophin receptor (p75NTR), often associated with pro-apoptotic signals and receptor modulation [39]. During MASLD progression, NGF presents a molecular conflict in the liver. While NGF/TrkA signaling provides essential protection for hepatocytes against oxidative stress and inflammation, activation of p75NTR by NGF or pro-NGF drives lipogenesis [39]. This pathway involves a p38 MAPK-caspase-2-SREBP2 cascade that promotes de novo lipogenesis and lipid retention, directly contributing to the steatotic phenotype. Simultaneously, NGF plays a protective role within the cardiovascular system, which is often under stress in MASLD patients. NGF functions as a pro-survival factor for cardiomyocytes and endothelial cells via the PI3K/Akt pathway, providing vital cardioprotection by excluding forkhead box O (FoxO) transcription factors from the nucleus to prevent apoptosis, maintaining vascular homeostasis through stimulated angiogenesis and nitric oxide production, and driving the reparative neovascularization response following myocardial injury that is often impaired in metabolic syndromes [40]. As MASLD progresses toward advanced fibrosis or cirrhosis, the role of NGF in the liver-heart axis may become maladaptive. Chronically elevated circulating NGF can trigger excessive sympathetic nerve sprouting in the heart [41]. This hyperinnervation creates a pro-arrhythmogenic environment, increasing the risk of lethal ventricular tachyarrhythmias and sudden cardiac death. Consequently, patients with MASLD experience a significantly higher incidence of lethal arrhythmias [42].

### Liver-Kidney Axis in MASLD

MASLD is increasingly recognized as a complex metabolic condition that can lead to serious complications beyond the liver. Due to the unique hepatorenal relationship, a metabolically distressed liver can support the development of chronic kidney disease (CKD) [2]. CKD is clinically associated with decreased estimated glomerular filtration rate (eGFR) and albuminuria, which are independent predictors of adverse kidney, cardiovascular, and mortality outcomes. Although CKD and MASLD often co-occur with other metabolic risk factors such as T2DM, obesity, and hypertension, several studies have observed a relationship between the two conditions after controlling for these additional metabolic risks [43]. The interplay between MASLD and CKD involves myriad metabolic pathways driven by a chronic low-grade inflammatory environment induced by intrahepatic lipid accumulation. This inflammatory state leads to the release of pro-inflammatory cytokines, and oxidative stress in tissues contributes to renal tubular damage.

The progression of MASLD may be facilitated by crosstalk between adipokines and hepatokines, such as fetuin-A and FGF2. While the protective adipokine adiponectin is often released during metabolic disease, abnormal release of fetuin-A in response to stress can disrupt insulin signaling, glucose and lipid metabolism, continued inflammation, and lipotoxicity, which contribute to development and progression of MASLD. As Fetuin-A production increases in hepatocytes, the hepatokine activates TLR4, leading to production of inflammatory cytokines [44]. Fetuin-A also inhibits insulin receptor tyrosine kinase activity, which promotes systemic insulin resistance. In addition to the inflammatory, insulin-resistant environment, this hepatokine decreases nitric oxide bioavailability and contributes to glomerular endothelial injury. In conjunction with feutin-A, the hepatokines Selenoprotein P, ANGPTL8, FGL1, and FGF21 promote insulin resistance and advance the development of CKD in MASLD [45]. As insulin resistance increases, renal dynamics are disrupted by sodium retention, leading to hypertension. For example, increased blood glucose levels due to insulin resistance can lead to increased proximal reabsorption of sodium decreasing sodium delivery to the macula densa and promoting hyperfiltration [46]. Uncontrolled hypertension can damage the delicate renal filtration system, thus leading to decreases in eGFR and increases in albuminuria.

In addition to the presence of MASLD and its effect on CKD risk, the severity of the liver dysfunction may play an important role in determining CKD severity. Studies have determined that patients with advanced hepatic dysfunction, characterized by steatohepatitis or advanced hepatic fibrosis, have an increased prevalence of CKD as compared to metabolically challenged, non-MASLD counterparts [47]. However, this study did not report a correlation between MASLD severity and CKD severity. High MASLD severity (fibrosis) has been correlated with increased CKD severity in two other studies [47, 48]. This observed relationship between MASLD and risk of CKD development illustrates a pathogenic link between progressive liver disease and metabolic-associated renal injury. These findings suggest that MASLD is an independent risk factor for CKD and highlight the importance of early detection and intervention in affected individuals through routine and advanced testing. Advanced screening for albuminuria and eGFR monitoring empowers timely intervention and long-term cardiorenal risk mitigation. Targeted determination of hepatokine profiles in MASLD patients at risk of CKD could also help to determine disease progression.

Fibroblast growth factor hormones 19 and 21 are hepatokines with distinct roles in the kidney [49]. Both hepatokines require the Beta Klotho protein as a cofactor to enable tissue-specific activity and receptor binding. FGF19 inhibits bile acid synthesis by blocking the expression of cholesterol 7-α-hydroxylase (CYP7A1) and plays a partial role in carbohydrate metabolism. FGF21 regulates triglyceride levels and mediates insulin-independent glucose uptake. FGF21 is also suggested to have anti-fibrotic effects and could be used to prevent the progression of CKD [50]. In CKD, both FGF19 and FGF21 are elevated. FGFs have a variety of benefits, including improved glucose tolerance, insulin sensitivity, lipid loss, and energy metabolism. Research shows significantly higher serum concentrations of both FGF19 and FGF21 in patients with CKD compared with individuals in the control, hemodialysis, and kidney transplantation groups [51, 52]. Additionally, a negative correlation between FGF19 and glomerular filtration rate was observed in patients with CKD, and higher FGF19 levels were associated with more advanced CKD [49]. The increased concentrations of both hepatokines in CKD may indicate a physiological defense mechanism in response to metabolic disorders exacerbated by kidney disease. Both the kidney transplant and haemodialysis groups reduced FGF19 and FGF21 concentrations in patients with CKD, demonstrating the effectiveness of available treatments for CKD [53]. The reported renoprotective associations of FGF21 analogs, include: improvements in GFR, increased urinary sodium excretion, and a decreased urine albumin-creatinine ratio, signifying that the risk of CKD has decreased [50]. Autophagy deficiency in kidney proximal tubular epithelial cells is another factor that induces FGF21 and can also disturb mitochondrial function. Autophagy is an important compensatory mechanism in CKD progression prevention by reducing kidney fibrosis. Research reveals that FGF21 is robustly activated in the context of autophagy irregularities and protects against worsening CKD during aging and obesity by compensating for and restoring autophagy and mitochondrial homeostasis [54].

In the context of MASLD, systemic BDNF levels are often diminished, reflecting a broader breakdown in metabolic and neuroendocrine signaling. MASLD is characterized by chronic low-grade inflammation, with elevated levels of cytokines such as TNF-α, IL-6, and TGF-β [55]. These pro-inflammatory factors contribute to a vicious cycle that impairs communication between the brain, liver, and kidney. The TGF-ß/BDNF axis has emerged as a central node in metabolic regulation. Under normal physiological conditions, the transcription factor CREB promotes cellular survival and metabolic homeostasis by driving BDNF gene transcription. This CREB-BDNF pathway acts as a neuroprotective and tissue-protective defense. In metabolic disease states like MASLD, however, the upregulation of pro-fibrotic, pro-inflammatory TGF-β actively impairs CREB activation and binding at the BDNF promoter, suppressing BDNF expression [56]*.* Consequently, the disruption of this CREB-driven TGF-β/BDNF signaling hub impairs protective mechanisms, accelerating both hepatic fibrogenesis and systemic neuro-metabolic dysfunction [57].

In the kidney, BDNF is critical for maintaining the structural integrity of the glomerular filtration barrier. BDNF and its receptor, TrkB, are expressed in renal podocytes, where they provide essential trophic support by stabilizing the actin cytoskeleton through the regulation of cofilin phosphorylation [58]. This protective pathway is compromised under diabetic and metabolic stress; specifically, the pathological upregulation of miR-155-5p directly suppresses downstream BDNF expression [59]. Consequently, the loss of BDNF signaling leaves podocytes vulnerable to reactive oxygen species accumulation and severe inflammatory cascades, accelerating podocyte injury and glomerular decline. As MASLD progresses to MASH, this vulnerability is compounded by systemic inflammation, leaving the kidney increasingly susceptible to oxidative stress and lipotoxicity, which contributes to the high prevalence of CKD in MASLD patients.

Shared pathophysiological pathways mediate the liver-kidney axis. The inflamed liver releases a secretome (Fig. 1), including increased levels of angiotensinogen, pro-inflammatory cytokines, and altered lipoproteins, that triggers the Renin–Angiotensin–Aldosterone System (RAAS) and promotes renal fibrosis [60]. Concurrently, this intrahepatic pathological environment is exacerbated by a disruption of hepatic chromatin accessibility via hnRNPU inactivation, which stimulates the expression of TrkB-T1 to promote downstream inflammatory signaling and hepatocyte cell death [29]*.* Ultimately, this cellular damage fuels the chronic hepatic secretome, reinforcing the destructive feedback loop between the liver and kidney.

Beyond its role in hepatic remodeling, NGF serves as a critical messenger in the liver-kidney axis. The kidney is highly sensitive to fluctuations in circulating and local NGF, which protect and promote the growth of renal sympathetic nerves [61]. In MASLD and related metabolic syndromes, elevated NGF release from the liver contributes to increased renal sympathetic nerve density [62]. This NGF-driven increase in renal nerve density creates a vicious cycle; the resulting sympathetic overactivity exacerbates hepatic metabolic dysfunction and systemic insulin resistance, further fueling the progression of MASLD [29]. Furthermore, because NGF influences systemic glucose and insulin homeostasis [63], its dysregulation in MASH creates an environment more conducive to diabetic kidney disease (DKD), often a comorbid condition in MASLD/MASH patients.

In the early or acute stages of hepatic injury, NGF functions as a potent cytoprotective agent. Rather than acting as a direct mitogen, NGF focuses on tissue stabilization through powerful anti-apoptotic and antioxidant effects [64]. Apoptosis in hepatocytes is critically governed by the balance between opposing proteins: the pro-survival protein Bcl-2 and the pro-apoptotic effector Bax. An elevated Bax/Bcl-2 ratio prompts mitochondrial outer membrane permeabilization, cytochrome c release, and programmed cell death. In the presence of the oxidative stress commonly seen in early MASLD, NGF activates the TrkA signaling pathway, stimulating Akt phosphorylation to restore the Bcl-2/Bax balance and safeguard the injured parenchyma. Furthermore, NGF mitigates local inflammation by suppressing pro-inflammatory cytokines (IL-1, IL-6, TNF-α) via the p38 MAPK pathway, providing a crucial buffer against the initial oxidative injury [65]*.*

As MASLD progresses to MASH, NGF influences systemic metabolic control by promoting insulin secretion in pancreatic islets [63], while simultaneously driving a pro-lipogenic mechanism within the liver via p75NTR. Activation of p75NTR triggers a p38 MAPK/caspase-2/caspase-3 cascade that cleaves and activates SREBP2, driving de novo lipogenesis and hepatic lipid accumulation [39, 66]. Conversely, during injury resolution, NGF acts as a paracrine death signal. Hepatocyte-derived NGF binds to p75NTR on activated hepatic stellate cells (HSCs), down-regulating NF-kB and sensitizing them to apoptosis. The efficacy of this fibrinolytic reset is governed by macrophage-derived MMP-7, which cleaves the pro-apoptotic precursor (pro-NGF) into its mature form during recovery [67]. The physiological and pathological outcomes of NGF/p75NTR signaling are highly pleiotropic, where whether the cascade exerts a beneficial or detrimental effect depends fundamentally on the microenvironmental context and the specific target cell type. The chronic transition toward pro-NGF/p75NTR signaling closely mirrors the deteriorating liver environment in metabolic disease. In the renal system, elevated concentrations of pro-NGF trigger apoptosis in renal tubular cells and catalyze interstitial fibrosis, effectively shifting the kidney from a capacity for adaptive repair to a state of chronic disease [68]*.*

### Pharmacological Therapy for MASLD

#### GLP-1 Receptor Agonists

Glucagon-like peptide-1 receptor agonists (GLP-1 RAs) have emerged as one of the most promising pharmacological therapies for MASLD, particularly in patients with coexisting obesity and T2DM. These substances have metabolic effects that are essential to the pathophysiology of MASLD, including glucose-dependent insulin secretion, delayed gastric emptying, appetite suppression, and substantial weight loss [69]. By improving insulin resistance, reducing de novo lipogenesis, and lowering systemic inflammation, GLP-1 RAs act to mechanistically reduce hepatic steatosis [70].

Their therapeutic role is strongly supported by clinical evidence. GLP-1 RAs effectively ameliorate liver steatosis, inflammation, and fibrosis in patients with MASLD/MASH, associated with decreases in body weight and liver enzyme levels [70]. Meta-analyses have also shown that GLP-1 RAs,such as liraglutide and semaglutide, improve liver histology and resolve steatohepatitis [71]. Semaglutide’s disease-modifying potential has been further supported by recent trials showing significant rates of MASH remission without fibrosis worsening [72]. It is unclear whether the beneficial effects of GLP-1 RAs are due to direct effects on hepatic metabolism or to the promotion of weight loss.

GLP-1 RAs exert systemic cardiorenal protective effects in patients with MASLD by targeting shared pathways of insulin resistance, lipotoxicity, and chronic inflammation. On the cardiovascular side, GLP-1 RAs reduce major adverse cardiovascular events, including myocardial infarction, stroke, and cardiovascular mortality, while lowering blood pressure, reducing EAT, and improving endothelial function and lipid profiles [73]. On the renal front, they confer nephroprotection by slowing the progression of albuminuria, preserving glomerular filtration rate, and attenuating oxidative stress and renal inflammation [74]. By simultaneously improving hepatic steatohepatitis and mitigating systemic vascular and renal damage, GLP-1 RAs address the primary drivers of morbidity and mortality across the interconnected liver–heart–kidney axis.

#### SGLT2 Inhibitors

Sodium–glucose cotransporter-2 (SGLT2) inhibitors, most commonly empagliflozin, dapagliflozin, and canagliflozin, originally developed for T2DM management, have demonstrated increasing utility in MASLD due to their favorable metabolic and hepatic effects. These medications reduce renal glucose reabsorption, thereby improving glycemic control, reducing insulin resistance, and promoting weight loss, all of which are major drivers of hepatic steatosis [75]. Furthermore, SGLT2 inhibitors directly affect the liver by reducing oxidative stress, inflammation, and lipotoxicity. Hirayama et al. Found that ipragliflozin dramatically lowers liver fat content and improve biochemical markers such as ALT and GGT in patients with MASLD [76]. The significance of SGLT2 inhibitors in lowering hepatic steatosis is also supported by a meta-analysis of randomized controlled trials that showed a significant decrease in liver fat content (standardized mean difference 0.73, *p* < 0.001) [77]. Furthermore, updated analyses suggest that SGLT2 inhibitors may slow the progression of liver fibrosis, although the results are modest and somewhat heterogeneous [75]. Mechanistically, SGLT2 inhibitors improve hepatic lipid metabolism by reducing lipogenesis, enhancing fatty acid oxidation, and modulating inflammatory cytokines, including TNF-α and IL-6 [75]. While these findings are promising, long-term histological outcomes and fibrosis regression remain areas of ongoing research, and current evidence supports their use primarily in patients with MASLD and T2DM.

SGLT2 inhibitors additionally exert a direct effect on systemic hemodynamics, reducing cardiac preload and afterload, restoring tubuloglomerular feedback, and preserving eGFR in patients with HFpEF and CKD [78]. A meta-analysis by McGuire et al. shows significant cardio-renal protective benefits in patients with T2D, with the greatest risk reductions observed for heart failure hospitalization (32% reduction; HR 0.68) and kidney disease progression (38% reduction; HR 0.62) [79].

#### PPAR Agonists

Peroxisome proliferator-activated receptors (PPARs) are a family of nuclear receptor proteins that act as transcription factors activating genes involved in regulating metabolism. Activation of PPARs has been demonstrated to have beneficial effects in metabolic conditions such as T2DM, obesity, and hyperlipidemia. One class of PPAR agonists acts by activating PPAR-γ. These drugs are generically referred to as thiazolidinediones, with the most common one being pioglitazone. Pioglitazone improves insulin sensitivity, redistributes lipids from the liver to adipose tissue, and reduces hepatic inflammation. Through these mechanisms, PPAR-γ agonists directly target the underlying metabolic dysfunction central to MASLD pathogenesis. Pioglitazone has been shown in several randomized controlled trials and meta-analyses to dramatically reduce hepatic steatosis, inflammation, and ballooning; some studies have also shown improvements in fibrosis [80]. The results of clinical trials demonstrate that pioglitazone reduced hepatic triglyceride levels, enhanced insulin sensitivity, and improved markers of NASH [81, 82]. Pioglitazone has also proven effective for MASLD when combined with other therapies, such as SGLT2 inhibitors [83]. Despite its efficacy, the clinical use of pioglitazone is complicated by adverse effects such as weight gain, fluid retention, and potential cardiovascular risks.

PPAR-α agonists are another class of potential treatments for MASLD. Activation of PPAR-α in the liver is associated with increased fatty acid oxidation and reductions in steatosis. Saroglitazar is a dual peroxisome proliferator-activated receptor alpha/gamma agonist that has been shown to improve MASLD markers in randomized controlled clinical trials [84, 85]. Saroglitazar has completed phase 3 and phase 4 clinical trials, demonstrating marked reductions in hepatic fat fraction and liver enzymes without significant fluid retention.

### Perspectives and Future Directions

The development of CVD is a significant risk for MASLD patients. Alterations in hepatic metabolism resulting in increased steatosis and insulin resistance can impact cardiac and renal function to promote CVD. Increased hepatic steatosis can alter hepatokine release, promoting cardiac and renal inflammation and fibrosis. The role of hepatokines such as BDNF, NGF, FGF19, and FGF21 in alterations in cardiac and kidney function in MASLD warrants further examination to explore new therapeutic opportunities for MASLD-associated CVD. The emergence of GLP-1 receptor agonists, SGLT2 inhibitors, and PPAR agonists also offers new hope as therapies for MASLD. However, the cardiovascular benefits of these drugs need further testing in MASLD patients. It is not known whether these therapies could offer direct benefits or benefit through changes in body weight or glucose levels. This will require future studies that control for the weight loss effects of these drugs so that direct effects can be examined.

## Key References

- Rinella ME, Sookoian S. From NAFLD to MASLD: updated naming and diagnosis criteria for fatty liver disease. J Lipid Res. 2024;65(1):100485.
  - ○ This article outlines the global consensus to transition from NAFLD to MASLD by defining the new diagnostic criteria.
- Moncho F, Benlloch S, Gorriz JL. The impact of metabolic dysfunction-associated steatotic liver disease (MASH) on the high risk of cardiovascular disease in CKD: interconnections and management. Clin Kidney J. 2025;18(9):sfaf260.
  - ○ This article details how overlapping metabolic pathways and systemic inflammation create a pathological link between hepatic steatosis, hypertension, and progressive renal decline.
- Huber Y, Hofmann L, Prochaska JH, Koeck T, Chalabi J, Pfeiffer N, et al. Incidence of major cardiovascular events in patients with metabolic dysfunction-associated steatotic liver disease in the general population. Eur J Heart Fail. 2025;27(11):2490–500.
  - ○ This paper highlights the relationship between MASLD and the risk of major cardiovascular events, emphasizing the crosstalk between the liver and the heart.
- Ono Y, Sakai K, Okamura T, Okada H, Obora A, Kojima T, et al. Metabolic dysfunction-associated steatotic liver disease (MASLD) can be a possible predictive factor of incident CKD: NAGALA cohort study. PLoS One. 2025;20(10):e0334720.
  - ○ This paper highlights the critical role of MASDLD as a predictive factor for CKD, emphasizing the interconnected pathophysiology with the liver-heart-kidney axis.
- Sonaglioni A, Cerini F, Fagiani V, Nicolosi GL, Rumi MG, Lombardo M, et al. Effect of Metabolic Dysfunction-Associated Steatotic Liver Disease (MASLD) on Left Ventricular Mechanics in Patients Without Overt Cardiac Disease: A Systematic Review and Meta-Analysis. J Clin Med. 2025;14(8).
  - ○ This paper provides quantitative evidence linking hepatic metabolic dysfunction to subclinical cardiac impairment. Focusing on patients without overt cardiovascular disease, it highlights that MASLD independently alters left ventricular function early in disease progression.
- Qu Y, Liu J, Li J, Shen S, Chen X, Tang H, et al. Association of abdominal adiposity, hepatic shear stiffness with subclinical left-ventricular remodeling evaluated by magnetic resonance in adults free of overt cardiovascular diseases: a prospective study. Cardiovasc Diabetol. 2023;22(1):99.
  - ○ This paper provides clinical imaging evidence which demonstrates that hepatic and visceral fat accumulation drives cardiac remodeling and impaired myocardial strain, even before overt CVD appears. This directly supports the idea that MASLD acts as an early, independent driver of cardiac dysfunction.
- Peng D, Yu Z, Wang M, Shi J, Sun L, Zhang Y, et al. Association of Metabolic Dysfunction-Associated Fatty Liver Disease With Left Ventricular Diastolic Function and Cardiac Morphology. Front Endocrinol (Lausanne). 2022;13:935,390.
  - ○ This article provides clinical echocardiographic evidence that MASLD is significantly linked to LV diastolic dysfunction and adverse remodeling. Furthermore, by showing that cardiac remodeling and diastolic impairment worsen progressively with increasing steatosis severity, the study directly substantiates the review's assertion of a dose-dependent pathological interaction between hepatic steatosis and cardiovascular dysfunction.
- Huning AR, Egypto Rosa VE, Piardi DS, Assis DVR, Ferreira TV, Griseli L, et al. Association Between Left Ventricular Global Longitudinal Strain and Hepatic Inflammation and Fibrosis in Metabolic Dysfunction-Associated Steatotic Liver Disease. Diagnostics (Basel). 2025;15(23).
  - ○ This study shows that left ventricular global longitudinal strain worsens as biopsy-confirmed hepatic inflammation and fibrosis progress, revealing early myocardial damage even with normal ejection fraction. This supports the review's concept that advancing liver disease severity drives a dose-dependent increase in subclinical cardiac dysfunction.
- Mantovani A, Petracca G, Csermely A, Beatrice G, Bonapace S, Rossi A, et al. Non-alcoholic fatty liver disease and risk of new-onset heart failure: an updated meta-analysis of about 11 million individuals. Gut. 2022.
  - ○ This meta-analysis provides data showing that MASLD independently increases the long-term risk of new-onset heart failure by 1.5-fold, with risk escalating alongside fibrosis severity. This validates the review's claim that MASLD directly drives clinical heart failure independent of cardiometabolic risk factors.
- Schulz A, Backhaus SJ, Lange T, Evertz R, Kutty S, Kowallick JT, et al. Impact of epicardial adipose tissue on cardiac function and morphology in patients with diastolic dysfunction. ESC Heart Fail. 2024;11(4):2013–22.
  - ○This paper provides evidence that increased epicardial adipose tissue is independently associated with regional myocardial function impairment, including diastolic abnormalties consistent with HFpEF-like dysfunction.
- Zhu Y, Ran Y, Fu T, Zheng X, Chen Y, Liang C, et al. Cardiomyocyte-derived BDNF restricts cardiac fibrosis by decreasing the activity of the TGF-beta/Smad2/3 pathway and increasing Smad7 expression. Front Cell Dev Biol. 2026;14:1786720.
  - ○ This paper provides experimental evidence that BDNF is crucial for cardiomyocyte survival and limits cardiac fibrosis by modulating fibroblast activity.
- Badmus OO, Kipp ZA, Bates EA, da Silva AA, Taylor LC, Martinez GJ, et al. Loss of hepatic PPARalpha in mice causes hypertension and cardiovascular disease. Am J Physiol Regul Integr Comp Physiol. 2023;325(1):R81–R95.
  - ○ This study reveals that hepatic PPARα deficiency leads to reduced circulating BDNF and impaired vascular TrkB signaling, directly substantiating the review’s proposed molecular pathway where disrupted liver- derived neurotrophic signaling promotes cardiovascular dysfunction along the cardiorenal-hepatic axis.
- Moosaie F, Mohammadi S, Saghazadeh A, Dehghani Firouzabadi F, Rezaei N. Brain-derived neurotrophic factor in diabetes mellitus: A systematic review and meta-analysis. PLoS One. 2023;18(2):e0268816.
  - ○ This paper provides meta-analytic evidence demonstrating that circulating BDNF levels are decreased in pateints with T2DM. Additionally, the paper highlights how BDNF/TrkB/CREB signaling suppresses hepatic gluconeogenesis and improves insulin sensitivity, supporting the idea that BDNF depletion drives systemic glucose dysregulation, worsening the metabolic burden on the heart and kidneys.
- Pezzino S, Luca T, Castorina M, Puleo S, Latteri S, Castorina S. Role of Perturbated Hemostasis in MASLD and Its Correlation with Adipokines. Life (Basel). 2024;14(1).
  - ○ This article demonstrates that MASLD promotes a pro-thrombotic and hypofibrinolytic state driven by elevated PAI-1, fibrinogen, and altered adipokines. This supports the review's explanation of how abnormal liver-derived clotting factors and fat inflammation accelerate cardiovascular disease.
- Simon TG, Ebrahimi F, Roelstraete B, Hagstrom H, Sundstrom J, Ludvigsson JF. Incident cardiac arrhythmias associated with metabolic dysfunction-associated steatotic liver disease: a nationwide histology cohort study. Cardiovasc Diabetol. 2023;22(1):343.
  - ○ This study supports the premise that advancing MASLD independently promotes a pro-arrhythmogenic environment.
- Beknazarova A, Kuvaeva V, Baltin M, Mutig K, Bobylev A. BDNF/TrkB Signaling in the Brain-Kidney Axis Under Functional Stress. Biology (Basel). 2026;15(9).
  - ○ This paper shows that BDNF/TrkB signaling protects kidney function by stabilizing the podocyte actin cytoskeleton to maintain the glomerular filtration barrier. This supports the review's explanation of how loss of BDNF in MASLD leads to podocyte injury and accelerates chronic kidney disease.
- Mkhize BC, Mosili P, Ngubane PS, Sibiya NH, Khathi A. Diet-induced prediabetes: Effects on the activity of the renin–angiotensin–aldosterone system in selected organs. J Diabetes Investig. 2022;13(5):768–80.
  - ○ This article demonstrates that diet-induced prediabetes triggers local RAAS upregulation, elevated tissue angiotensinogen, and increased oxidative stress across metabolic organs, including the liver and heart.
- Samario-Roman J, Larque C, Panico P, Ortiz-Huidobro RI, Velasco M, Escalona R, et al. NGF and Its Role in Immunoendocrine Communication during Metabolic Syndrome. Int J Mol Sci. 2023;24(3).
  - ○ This paper details how dysregulated NGF signaling contributes to metabolic syndrome by modulating glucose-stimulated insulin secretion, adipokine release, and sympathetic nervous system activity.
- Usta M, Cigremis Y, Ozen H. Nerve growth factor acts as a modulator on the p38 MAPK pathway in copper-induced liver damage. J Trace Elem Med Biol. 2025;90:127694.
  - ○ This article provides experimental evidence that NGF acts as a cytoprotective agent in the liver by suppressing oxidative stress, reducing apoptosis, and downregulating pro-inflammatory cytokines. The study substantiates the context-dependent role of NGF in MASLD.
- Yang L, Ruan J, Fang Y, Xu A. Effect of SGLT-2 inhibitors on liver fibrosis progression in patients with MASLD: an updated meta-analysis based on RCTs. Front Med (Lausanne). 2025;12:1667823.
- Kim G, Yu TY, Jee JH, Bae JC, Kang M, Kim JH. Association between nonalcoholic fatty liver disease and left ventricular diastolic dysfunction: A 7-year retrospective cohort study of 3,380 adults using serial echocardiography. Diabetes Metab. 2024;50(3):101534.
  - ○ This paper demonstrates that MASLD is independently associated with the development of left ventricular diastolic dysfunction.
- Genua I, Cusi K. Pharmacological Approaches to Nonalcoholic Fatty Liver Disease: Current and Future Therapies. Diabetes Spectr. 2024;37(1):48–58.
  - ○ This is a comprehensive review of pharmacological treatments for MASLD.

## Data Availability

No datasets were generated or analysed during the current study.
